# Childhood neglect and problematic smartphone use among chinese young adults: the mediating roles of peer attachment and fear of missing out

**DOI:** 10.1186/s40359-023-01326-9

**Published:** 2023-10-03

**Authors:** Yan Zhang, Ding-liang Tan, Bo Jiang, Ting-ting Lei

**Affiliations:** 1 School of Education Science, Jiangsu Second Normal University, Nanjing, 210013 China; 2 School of Education Science, Nanjing Normal University, Nanjing, 210097 China

**Keywords:** Childhood neglect, Peer attachment, Fear of missing out, Problematic smartphone use, Young adults

## Abstract

**Background:**

Research has revealed that childhood neglect may be a risk factor for problematic smartphone use among young adults in China. However, few studies have examined the mediating roles of peer attachment and the fear of missing out in the relationship between childhood neglect and problematic smartphone use. To fill this gap, the present study proposes a multiple mediation model to understand the relationships among childhood neglect, peer attachment, fear of missing out, and problematic smartphone use among young adults.

**Methods:**

A total of 869 young adults in China completed questionnaires for evaluating different levels of the relationships between childhood neglect, peer attachment, the fear of missing out, and problematic smartphone use. The collected data were analyzed using SPSS 23.0 and MPLUS8.3.

**Results:**

The results showed that childhood neglect was positively associated with problematic smartphone use among young adults in China. Moreover, peer attachment and the fear of missing out had partial mediating effects as well as sequential mediating effects in the relationship between childhood neglect and problematic smartphone use among young adults.

**Conclusion:**

Based on these findings, peer attachment and the fear of missing out, as mediators, could be considered proximal factors affecting problematic smartphone use among young adults. These findings broaden our understanding of the psychological processes that underlie the association between childhood neglect and problematic smartphone use and afford practical guidance on reducing the risks associated with problematic smartphone use.

## Introduction

Every technological revolution results in significant changes throughout human society. The current information age, which is significantly characterized by rapid developments in mobile technology, has resulted in profound changes, affecting humankind on a global scale. On the one hand, mobile Internet has gradually become a crucial driving force of social development, thereby making social life highly convenient. On the other hand, it has continuously penetrated people’s daily lives, and the use of mobile devices, such as smartphones, has occupied almost all of people’s fragmented time. These aspects of mobile technology have significantly increased the risk of problematic smartphone use. According to the China Internet Network Information Center (2022), as of December 2021, the number of mobile Internet users in China was 1.029 billion, with young adults making up a significantly high proportion of such users (19.7%) [1]. Problematic smartphone use is associated with various negative consequences among young adults [2], such as deteriorations in the quality of sleep [3] and mental or physical problems [4–6].

### Childhood neglect and problematic smartphone use

The family environment has a direct and lasting impact on psychological development among different individuals. Childhood neglect is a crucial factor affecting psychological development among individuals in family environments. Childhood neglect involves the long-term negligence of children’s basic needs, pertaining to their emotions, education, and physical health, among other factors, by parents or guardians [7]. Currently, owing to rapid developments in various social and economic environments, childhood emotional neglect is a prevalent phenomenon. In a survey conducted by Sun et al. (2019), the results showed that 76.46% of the adolescent participants believed that they had suffered psychological neglect during their childhood [8].

Childhood neglect has a sustained negative impact on both physical and mental health among various individuals. The Interaction of Person-Affect-Cognition-Execution (I-PACE) model posits that negative early childhood experiences, such as childhood neglect, are distal risk factors that may make individuals vulnerable to develop an addictive behavior in particular [9]. Attachment theory posits that individuals who lack parent-child interaction during the critical period of development form unsafe attachment styles with their parents and other caregivers, thereby affecting their interpersonal interaction behaviors with other individuals in the future [10]. Based on attachment theory, young adults who suffered from neglect during their childhood often find it difficult to establish close emotional connections with the people around them, and they cannot get a sufficient sense of security from other individuals. Individuals with insecure attachments tend to use smartphones to satisfy their emotional needs and reinforce their sense of security, and this aspect results in problematic smartphone use [11]. Empirical studies have also established that childhood neglect is a crucial factor influencing the levels of problematic smartphone use among young adults [12]. According to a recent longitudinal study, childhood emotional neglect has a one-way causal relationship with problematic smartphone use among adolescents in China [13]. Therefore, in this study, we hypothesize that childhood neglect may have a significantly positive predictive effect on problematic smartphone use among young adults (***Hypothesis 1***).

Few studies have been conducted on the inherent mechanism of childhood neglect in young adults’ problematic smartphone use. In particular, the underlying multiple mediating mechanisms involved in this association between childhood neglect and young adults’ problematic smartphone use are largely unknown. To reveal these mechanisms, the present research constructed a multiple mediating model to examine the mediating roles of peer attachment and fear of missing out in the relation between childhood neglect and young adults’ problematic smartphone use from the perspectives of attachment theory and Interaction of Person-Affect-Cognition-Execution (I-PACE) model.

### Mediating role of the fear of missing out

Among individuals, the fear of missing out refers to a type of diffused anxiety resulting from the perception that other people are having novel experiences, having more fun, or living better lives in comparison [14]. In modern contexts, the fear of missing out has become a common psychological phenomenon among people. According to a recent study, approximately 78.3% of young adults in China experience the fear of missing out [15]. Based on the I-PACE model, when being confronted with abnormal mood, such as the fear of missing out, an urge to regulate the experienced mood may develop, which is considered as an important factor within the development of Internet-use disorders. As tools for observing the external world, smartphones make it significantly easy for individuals to understand external changes so as to regulate the experienced mood [16]. Once individuals understand the related external changes and regulate the experienced mood through smartphones, this behavior is rapidly exacerbated, and eventually, it gets out of control, thereby resulting in problematic smartphone use. Various empirical studies have also established that the fear of missing out is a vital risk factor for problematic smartphone use among young adults [12, 17]. Therefore, the fear of missing out may be considered as a proximal risk factor for problematic smartphone use among young adults.

Additionally, the interaction of person-affect-cognition-execution (I-PACE) model indicates that childhood experiences affect the way in which individuals form specific cognitive and emotional response patterns that affect self-inhibition ability and the desire for internet use, among other attributes, which eventually result in various types of internet-use disorders [9]. The experience of childhood neglect may lead young adults to develop a cognitive pattern of being neglected, where their psychological needs, especially relatedness, can’t be met. Meanwhile, the fear of missing out can be perceived as an expression of self-regulation obstruction among individuals [14]. According to the self-determination theory [18], effective self-regulation among individuals relies on meeting their three basic psychological needs: autonomy, competence, and relatedness. That is to say, when one’s basic psychological needs can’t be met, individuals will experience obstacles in self-regulation, resulting in the fear of missing out. Therefore, childhood neglect may be a risk factor that induces the fear of missing out among young adults. Empirical studies have also established that childhood neglect is also a crucial factor affecting the fear of missing out among young adults [12]. To alleviate the fear of missing out, young adults often pay attention to and track the news and life trajectories of their peers through smartphones at any time. Eventually, such behaviors result in problematic smartphone use. Therefore, we hypothesize that the fear of missing out may mediate the relationship between childhood neglect and problematic smartphone use among young adults (***Hypothesis 2***).

### Mediating role of peer attachment

Various recent studies employ the attachment theory to explore the relationship between peer attachment and problematic smartphone use among young adults [19, 20]. The development and strengthening of attachment-related bonds between children and their parents begin during infancy. As adolescents mature into young adults, the communication and interaction levels between them and their peers increase gradually, thereby resulting in the increased importance of peer attachment. Among young adults, the importance of peer attachment is significantly associated with the development of additional social relationships that can ensure support and encouragement in the face of the transitions and challenges associated with life [21].

Perceived social support is considered, to a large extent, as a by-product of attachment style, and individuals with insecure attachment endorse the lower of perceived social support [22]. Meanwhile, individuals with insecure attachment may be sensitive to fluctuation in their social environment and to negative attitudes and behaviors of others, which might lead them to experience states of loneliness [23]. That is, individuals with insecure peer attachment can’t establish positive and intimate interpersonal relationships in real life, which leads to negative social cognition such as perceived lack of social support and loneliness. Based on the Interaction of Person-Affect-Cognition-Execution (I-PACE) model, negative social cognition, as person’s core characteristic, is associated with specific Internet-use disorder. Young adults with low levels of peer attachment cannot establish positive and intimate interpersonal relationships in real life. Such individuals often turn to the virtual online world to satisfy and compensate for their psychological needs for interpersonal communication, thereby increasing the risk of problematic smartphone use. Additionally, various empirical studies have established that peer attachment has a significantly negative predictive effect on problematic smartphone use among young adults [21, 24].

The internal working model of attachment further postulates that early parent-child interaction significantly affects individuals’ future cognitive, emotional, and interpersonal interactions, as well as other behavioral patterns [25]. Young adults who have experienced childhood neglect often form unsafe attachments with their parents, and this attribute affects their interpersonal interaction with others in the future, thereby resulting in insecure peer attachments among such individuals. Young adults with insecure peer attachment often seek to gratify their psychological needs of interpersonal communication using smartphones. Over time, this behavior results in problematic smartphone use among such individuals. Based on the above, we hypothesize that peer attachment may mediate the relationship between childhood neglect and problematic smartphone use among young adults (***Hypothesis 3***).

Although there is no study that directly explores the relationship between peer attachment and the fear of missing out, a recent study established that anxious attachment has a significantly positive predictive effect on the fear of missing out among young adults [26]. Young adults with low levels of peer attachment often find it difficult to form a safe attachment relationship with their peers. Therefore, such individuals are highly likely to worry, and they fear missing out on news relevant to their peers, and this attribute results in the emergence of the fear of missing out. To eliminate the fear of missing out in this case, such individuals always use their smartphones to obtain the relevant dynamics of their peers’ lives, thereby inducing the risk of problematic smartphone use. In this study, we hypothesized that peer attachment and the fear of missing out may have sequential mediating effects on the relationship between childhood neglect and problematic smartphone use among young adults (***Hypothesis 4***).

### The current study

**Fig. 1:**
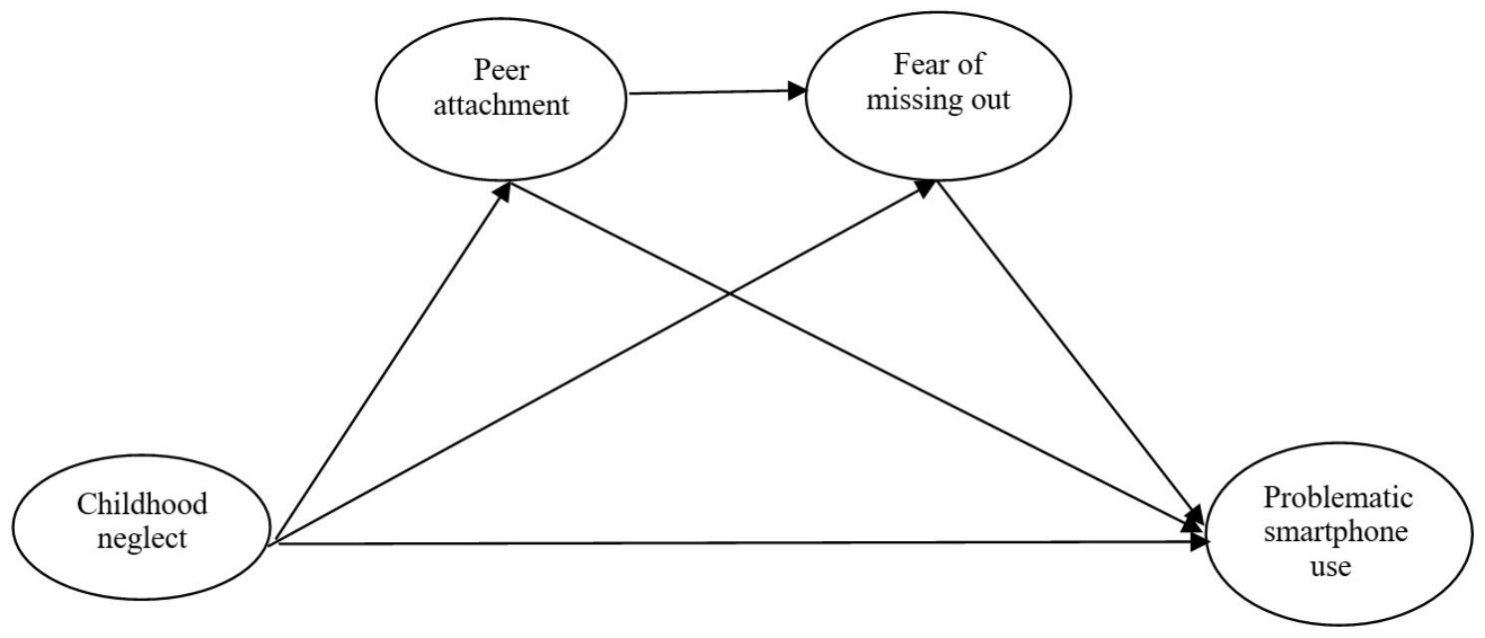
Conceptual model

In this study, based on theoretical support from previous studies and the explanations presented above, we predicted that young adults who suffer from childhood neglect form insecure attachments with their peers, and thus, they experience high levels of the fear of missing out. As a result, such individuals are highly likely to engage in problematic smartphone use. Because the association between these variables was considered to have a significant influence on the levels of problematic smartphone use among young adults, it was determined that peer attachment and the fear of missing out mediate the relationship between childhood neglect and problematic smartphone use among such individuals (Fig. 1).

We proposed a structural model for explaining the relationship between childhood neglect and problematic smartphone use, with the mediators being peer attachment and the fear of missing out. In other words, based on the model proposed and explored in this study, we attempted to answer three questions, as follows: (a) Does the fear of missing out mediate the relationship between childhood neglect and problematic smartphone use? (b) Does peer attachment mediate the relationship between childhood neglect and problematic smartphone use? and (c) Do peer attachment and the fear of missing out have sequential mediating effects on the relationship between childhood neglect and problematic smartphone use among young adults? It is possible to provide further insight into the studies related to problematic smartphone use by examining to what extent and the way in which these main factors, which were selected from the environmental and individual sources of problematic smartphone use, can be used to predict problematic smartphone use among young adults.

## Methods

### Participants

In this study, based on cluster sampling, a total of 912 young adults were approached to participate in the survey, and 869 (95.28%) responded. The measure of deleting cases with missing values was adopted to handle missing data. A total of 438 college students from two large public research universities located in Jiangsu Province, Eastern China and 431 college students from two large public research universities located in Shanxi Province, Western China participated in this study. The sample size involved in this study was deemed appropriate for obtaining a Cronbach’s α coefficient of 0.05 based on calculations carried out through G*Power. The sample population’s mean age was 20.48 years (SD = 1.12, with an age range of 19–22 years). The sample comprised 414 males (47.64%) and 455 females (52.36%). A total of 348 (40.04%) participants were from urban families in China, and 521 (59.96%) participants were from rural families in China. Among the participants, 225 were freshmen (25.89%), 213 were sophomores (24.51%), 219 were junior students (25.20), and 212 were senior students (24.40%).

### Measurements

#### Childhood neglect

The Chinese version of the Childhood Neglect Scale [27] was used in this study. This scale comprises 17 items that assess three factors of childhood neglect: emotional neglect, educational neglect, and physical/supervisory neglect. The participants rated these items using a five-point scale (1 = never, 5 = always). Higher scores reflected higher levels of childhood neglect. In this study, each subscale had good internal consistency: emotional neglect (α = 0.77), educational neglect (α = 0.82), and physical/supervisory neglect (α = 0.86). And Cronbach’s α for the CNS was 0.88. Likewise, confirmatory factor analysis indicated that the three-factor model demonstrated a good fit to the data (*x*^*2*^*/df* = 4.29, CFI = 0.91, TLI = 0.94, SRMR = 0.06, and RMSEA = 0.05).

#### Peer attachment

In this study, the short version of Inventory of Parent and Peer Attachment [28] was used to measure the levels of peer attachment. This scale assesses three factors related to peer attachment: trust, communication, and alienation. The participants were instructed to rate the extent to which each item was descriptive of peer attachment. The scale included 12 items, which were rated by the participants on a five-point scale (1 = never, 5 = always). Higher scores indicated an increased tendency for attachment towards peers. This measure was used successfully among Chinese sample with great reliability and validity [28]. In this study, each subscale had good internal consistency: mutual trust (α = 0.78), communication (α = 0.80), and alienation (α = 0.81). Cronbach’s α for the IPPA was 0.84. Confirmatory factor analysis further indicated that the three-factor model demonstrated a good fit to the data (*x*^*2*^*/df* = 4.76, CFI = 0.90, TLI = 0.92, SRMR = 0.06, and RMSEA = 0.06).

#### Fear of missing out

The Chinese version of Fear of Missing Out Scale [29] was used in this study. The scale comprises eight items for assessing two factors related to the fear of missing out: fear of missing out on information and fear of missing out on situation. The participants rated these items on a five-point scale (1 = not at all true to 5 = extremely true). This measure demonstrated satisfactory reliability and validity among Chinese young adults [29]. In this study, both subscales had good internal consistency: fear of missing out on information (α = 0.75) and fear of missing out on situation (α = 0.82). Cronbach’s α for the FoMOS was 0.83. Likewise, confirmatory factor analysis indicated that the two-factor model demonstrated a good fit to the data (*x*^*2*^*/df* = 5.14, CFI = 0.92, TLI = 0.95, SRMR = 0.06, and RMSEA = 0.05).

#### Problematic smartphone use

In this study, the Mobile Phone Addiction Index (MPAI) [30] was used to measure the levels of problematic smartphone use. This index comprises 17 items for assessing four factors related to problematic smartphone use: anxiety and feeling lost, inability to control cravings, withdrawal and escape, and productivity loss. The participants rated these items on a five-point scale (1 = never, 5 = always). Cronbach’s α for the MPAI was 0.91, and in this study, the Cronbach’s α was 0.87. Higher scores indicated higher levels of problematic smartphone use. Good psychometric properties were reported among Chinese young adults [20, 30]. In this study, each subscale had good internal consistency: anxiety and feeling lost (α = 0.85), inability to control cravings (α = 0.82), withdrawal and escape (α = 0.84), and productivity loss (α = 0.81). Cronbach’s α for the MPAI was 0.91. Confirmatory factor analysis further indicated that the four-factor model demonstrated a good fit to the data (*x*^*2*^*/df* = 4.18, CFI = 0.93, TLI = 0.95, SRMR = 0.05, and RMSEA = 0.04).

### Procedure

Participants were recruited from four universities located in the two cities of Nanjing and Xian, China. The survey was completed in their classrooms during school hours. The quantitative data were collected by administering the following scales to these university students in their classrooms during school hours: the Chinese version of the Childhood Neglect Scale, the Inventory of Parent and Peer Attachment, the Fear of Missing Out Scale, and the Mobile Phone Addiction Index. Each class was provided with one trained psychology graduate student who was responsible for explaining the questions and helping students with answering the questionnaires. All the participants volunteered to take part in this study and completed a 30-minute questionnaire packet using a paper–pencil format. The questionnaires were anonymous.

### Statistical analysis

The collected data were analyzed using SPSS 23.0 and MPLUS8.3. First, we used descriptive statistics, including mean and standard deviation, to examine the characteristics of the measured variables. Second, we used Pearson’s correlation to evaluate the correlations between the variables. Finally, through structural equation modeling (SEM), we evaluated the performance of the hypothesized multiple mediation model involving the influence of peer attachment and the fear of missing out in the relationship between childhood neglect and problematic smartphone use among young adults. This study adopted the criteria for acceptable model fit [31], as follows: *x*^*2*^*/df* < 5, CFI and TLI > 0.90, RMSEA < 0.08, and SRMR ≤ 0.05. Childhood neglect, peer attachment, the fear of missing out, and problematic smartphone use were all treated as latent variables. The bootstrap method was used to evaluate the significance of all the effects. The robust standard error and confidence interval of parameter estimation were obtained by constructing 1000 bootstrap samples, with a sample size of 869 people. If the confidence interval did not contain zero, it indicated statistical significance [32].

## Results

### Descriptive statistics and correlations of the main study variables

Table 1 lists the results of the descriptive statistics and correlation analyses between variables. Gender was positively correlated with peer attachment (*p* < 0.01). Grade was positively correlated with peer attachment and problematic smartphone use (*p* < 0.05). Hailing from urban and rural areas was positively correlated with problematic smartphone use (*p* < 0.05). Childhood neglect was positively correlated with the fear of missing out and problematic smartphone use (*p* < 0.001), but it was negatively correlated with peer attachment (*p* < 0.001). Peer attachment was negatively correlated with the fear of missing out and problematic smartphone use (*p* < 0.001). The fear of missing out was positively correlated with problematic smartphone use (*p* < 0.001).

**Table 1:** Descriptive Statistics and Correlations of Main Study Variables

Variables	1	2	3	4	5	6	7
1.Gender	1						
2.Grade	0.04	1					
3. Hailing from urban and rural areas	0.03	0.01	1				
4. Childhood neglect	0.05	0.05	0.01	1			
5. Peer attachment	0.11^**^	0.07^*^	0.01	-0.43^***^	1		
6. Fear of missing out	0.04	0.05	0.04	0.31^***^	-0.25^***^	1	
7. Problematic smartphone use	0.05	0.07^*^	0.08^*^	0.28^***^	-0.27^***^	0.53^***^	1
*Mean*	0.52	2.48	0.60	2.37	3.53	2.89	3.53
*SD*	0.50	1.11	0.49	0.66	0.49	0.77	0.78

Note: *N* = 869. Gender: 0, male; 1, female. Hailing from urban and rural area: 0, Hailing from urban areas; 1, Hailing from rural areas. ^*^p < 0.05, ^**^p < 0.01, ^***^p < 0.001

### Measurement model testing

Confirmatory factor analysis was conducted to validate the factor structure of the study variables. The hypothesized four-factor measurement model provided a good fit, *x*^*2*^*/df* = 4.36, *p* < 0.001, CFI = 0.93, TLI = 0.95, and RMSEA = 0.05. In the measurement model, all factor loadings for the indicators of the four latent variables were significant (*p* < 0.001) and greater than 0.6, thereby indicating that all the latent factors were well represented by their respective indicators.

### Hypothesized model testing

**Fig. 2:**
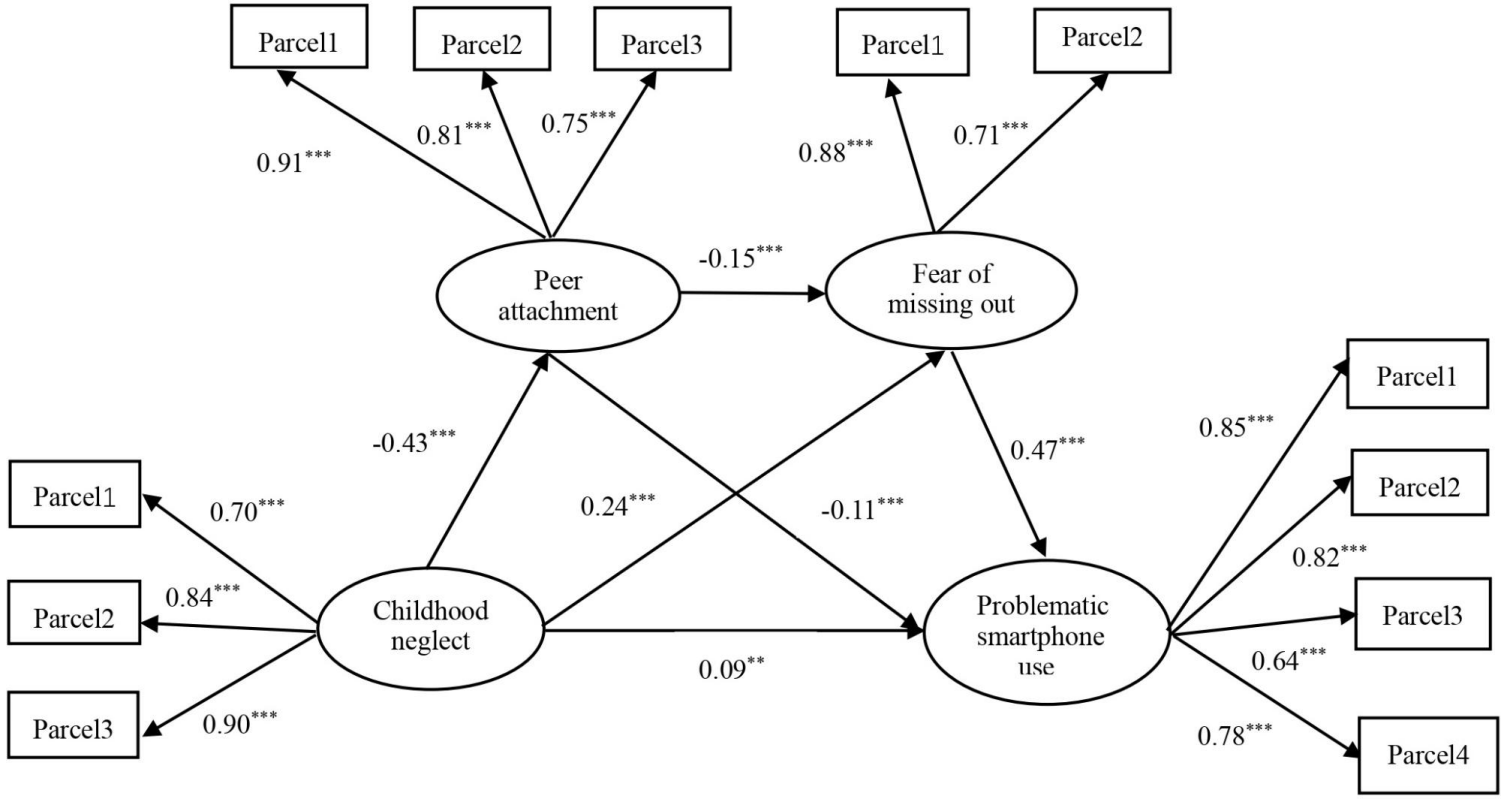
Results of the SEM model among the key variables. Note: ^**^p < 0.01, ^***^p < 0.001.

The hypothesized model was fitted with childhood neglect as an exogenous variable, peer attachment and the fear of missing out as mediators, and problematic smartphone use as the outcome variable. The effects of grade and urban or rural students were controlled on problematic smartphone use, whereas the effects of gender and grade were controlled on peer attachment. As shown in Fig. 2, the performance of the multiple-mediation model was also evaluated using SEM (*R*^2^ = 0.30, *p* < 0.001), and it fit the data well (*x*^*2*^*/df* = 5.37, CFI = 0.91, TLI = 0.94, SRMR = 0.05, and RMSEA = 0.06).

Childhood neglect was positively correlated with problematic smartphone use (*b* = 0.28, *p* < 0.001) before accounting for the mediation variable. In this study, the accuracy of Hypothesis 1 was tested first. After the mediator was considered, the direct variable was weakened (*b* = 0.09, *p* < 0.01). In the mediator model, childhood neglect was positively correlated with the fear of missing out (*b* = 0.24, *p* < 0.001), and the fear of missing out was positively associated with problematic smartphone use (*b* = 0.47, *p* < 0.001). Consistent with Hypothesis 2, the results of the bootstrapping analyses suggested that the fear of missing out partially mediated the relationship between childhood neglect and problematic smartphone use (indirect effect = 0.11, *Boot LLCI =* 0.08, *Boot ULCI =* 0.16). Additionally, childhood neglect was negatively correlated with peer attachment (*b* = -0.43, *p* < 0.001), and peer attachment was negatively associated with problematic smartphone use (*b* = -0.11, *p* < 0.001). Consistent with Hypothesis 3, the results of the bootstrapping analyses suggested that peer attachment partially mediated the relationship between childhood neglect and problematic smartphone use (indirect effect = 0.05, *Boot LLCI =* 0.02, *Boot ULCI =* 0.07). Furthermore, consistent with Hypothesis 4, the results of the bootstrapping analyses suggested that peer attachment and the fear of missing out had multiple mediating effects on the relationship between childhood neglect and problematic smartphone use among young adults (indirect effect = 0.03, *Boot LLCI =* 0.02, *Boot ULCI =* 0.05). The direct and indirect effects of the variables are listed in Table 2.

**Table 2:** Total, direct, and indirect effects of the pathways tested

*Pathway*	*Standardized Estimate*	*Boot SE*	*Boot LLCI*	*Boot ULCI*
Total	0.28	0.03	0.22	0.35
Direct effect: CN → PSU	0.09	0.04	0.03	0.17
Specific indirect effect: CN → PA → PSU	0.05	0.01	0.02	0.07
Specific indirect effect: CN → FoMO → PSU	0.11	0.02	0.08	0.16
Specific indirect effect: CN → PA → FoMO → PSU	0.03	0.01	0.02	0.05

Note: CN: childhood neglect, PA: peer attachment, FoMO: fear of missing out, PSU: problematic smartphone use

## Discussion

In this study, we constructed a serial mediation model for examining the mediating roles of peer attachment and the fear of missing out in the relationship between childhood neglect and problematic smartphone use among young adults. Generally, the results of this study confirmed the hypothesized relationships and indicated that childhood neglect is positively associated with problematic smartphone use and that this association is partially and sequentially mediated by peer attachment and the fear of missing out.

As expected, Hypothesis 1 was supported in that childhood neglect might be positively associated with problematic smartphone use among young adults. Therefore, the role of childhood neglect as a potential antecedent of problematic smartphone use was confirmed. This result is similar to those of previous studies on problematic smartphone use among young adults [12, 33]. Young adults suffering from childhood neglect cannot establish a secure attachment with others. Therefore, such individuals often use smartphones to satisfy their emotional and security needs. Over time, this behavior results in problematic smartphone use among such individuals. This result further verified the accuracy of the uses and gratifications theory [34].

This study indicated that the fear of missing out might partially mediate the relationship between childhood neglect and problematic smartphone use among young adults. This result is consistent with the previous research among young adults [12]. Essentially, young adults suffering from childhood neglect have high levels of the fear of missing out, and thus, they are likely to engage in problematic smartphone use. This result further enriched the content of the I-PACE model [9]. Young adults suffering from childhood neglect are those that have lived in family environments lacking safety and warmth since childhood. Therefore, such individuals often form a cognitive model that regards “being ignored” as a threat to their life or safety. This cognitive model results in such individuals hoping to participate in others’ lives all the time. This requires them to always pay attention to the dynamics of their friends’ lives. Otherwise, such individuals will have the cognition of “being ignored,” and this attribute results in the emergence of the fear of missing out. Such young adults can alleviate their fears and anxieties only when they know the whereabouts of others or what others are doing. Therefore, to alleviate the fear of missing out, such young adults frequently use their smartphones to pay attention to and track the news and life trajectories of their peers at any time. This behavior can temporarily satisfy the psychological needs associated with the sense of belonging and interpersonal attachments, and it can alleviate the fear of missing out among such individuals. However, such behavior makes such individuals addicted to smartphones, and as a result, they cannot extricate themselves. Eventually, this behavior results in problematic smartphone use.

This study also indicated that peer attachment might partially mediate the relationship between childhood neglect and problematic smartphone use among young adults. This result is similar to those of previous studies on problematic smartphone use among Korean youth [21, 35]. In other words, young adults suffering from childhood neglect form insecure attachments with their peers, and thus, they are highly likely to engage in problematic smartphone use. Young adults suffering from childhood neglect often form insecure attachments with their parents, and this attribute affects their future interpersonal interactions, thereby resulting in insecure attachments with their peers [36]. Based on the uses and gratifications theory, young adults with insecure peer attachment often use smartphones to satisfy and compensate for their psychological needs, as they pertain to interpersonal communication and attachment. Parent and Shapka (2020) explored the relationship between young adults and their smartphones based on the attachment theory [19]. According to their perspective, young adults develop attachment relationships with their smartphones, and they use these devices to satisfy their attachment needs. Young people who suffer from childhood neglect form unsafe attachments with their peers, and in order to protect themselves from physical and psychological harm, smartphones might be used as a source of safety, comfort, and security. Moreover, once smartphones, as objects that can enable attachment, are associated with positive outcomes, engaging with them provides a feeling of comfort in general, even in the absence of a stressor [19]. Therefore, peer attachment had partial mediating effect in the relationship between childhood neglect and problematic smartphone use among young adults.

Moreover, in this study, we established that childhood neglect might positively predict problematic smartphone use through the sequential mediating effects of peer attachment and the fear of missing out. In other words, young adults suffering from childhood neglect form insecure attachments with their peers, and thus, they have high levels of the fear of missing out. As a result, such individuals are highly likely to engage in problematic smartphone use. This research finding is a necessary supplement to the previous research [12]. Young adults with insecure peer attachments find it difficult to establish positive and close interpersonal relationships throughout their lives, and this attribute results in their inability to satisfy their needs, as they pertain to relatedness. As an expression of emotional self-regulation obstruction among individuals, the fear of missing out will occur when young adults cannot satisfy their needs, as they pertain to relatedness. Previous empirical studies have also established that social needs, such as the need to belong and need for popularity, are potential antecedents of the fear of missing out [37]. Therefore, peer attachment is negatively related to the fear of missing out among young adults. This result is similar to a previous empirical study, which had established that anxiety attachment is a crucial factor influencing the levels of Fear of Missing Out [26]. Meanwhile, compared with western cultures, East Asian cultures, including China, encourage people to suppress emotions, discourage the expression of individual emotions in person, and try to avoid direct expression of love and hate in interpersonal interaction. Previous studies have also pointed out that Western culture encourages and supports individual emotional expression, while Chinese culture suppresses the free expression of individual emotions, believing that emotional expression will damage interpersonal harmony [38, 39]. Therefore, young Chinese adults, when their insecure peer attachment leads to problems in peer communication, do not directly express their emotions and solve problems in person, leading to the fear of missing out. In order to alleviate the fear of missing out, they are more inclined to use their smartphones to pay attention to and track the news and life trajectories of their peers at any time. As such, childhood neglect can decrease the level of peer attachment, thereby resulting in the fear of missing out, which in turn increases the risk of problematic smartphone use among young adults. The results of this study go beyond the findings of previous studies because they demonstrate that childhood neglect may positively predict problematic smartphone use through the sequential mediating effects of peer attachment and the fear of missing out.

### Theoretical and practical implications

The model proposed and evaluated in this study demonstrated that childhood neglect is positively related to problematic smartphone use among young adults. Additionally, peer attachment and the fear of missing had independent mediating effects as well as multiple mediating effects on the relationship between childhood neglect and problematic smartphone use among young adults. To the best of our knowledge, this study is the first to focus on the mediating roles of peer attachment and the fear of missing out in the relationship between childhood neglect and problematic smartphone use among young adults in China from the perspectives of the attachment theory and I-PACE model. This study’s conclusion provides a novel perspective on the relationship between childhood neglect and problematic smartphone use among young adults in China. Another contribution of this study is that it explores the mediating role of peer attachment in the relationship between childhood neglect and problematic smartphone use among young adults in China. The findings of this study contribute to existing literature about the attachment theory and its role in understanding problematic smartphone use among young adults.

The results of this study have significant implications in the development of prevention and intervention strategies aimed at addressing the issue of problematic smartphone use among young adults in China. In other words, although the experiences of young people suffering from childhood neglect cannot be changed, the probability of problematic smartphone use among such individuals can be reduced by improving the levels of peer attachment and reducing the levels of the fear of missing out. First, the results of this study suggest that to reduce the risk of problematic smartphone use, young adults should increase their levels of peer attachment. Educators can improve the levels of peer attachment among young adults through various intervention strategies, such as cognitive behavioral play therapy. According to a recent study, group intervention approaches based on cognitive behavioral play therapy significantly improve the quality of attachment among young adults that have experienced childhood trauma [40]. The study also established that the risk of problematic smartphone use can be reduced by reducing the levels of the fear of missing out. Although there are few experimental studies on interventions targeting the fear of missing out, empirical studies on this subject have established that the satisfaction of social psychological needs, such as belonging, can effectively reduce the levels of the fear of missing out [37]. Therefore, young adults suffering from childhood neglect should be encouraged to increase their participation in social activities associated with real life situations to satisfy their social psychological needs. This approach can be used to reduce the levels of the fear of missing out and further avoid the risk of problematic smartphone use among such individuals.

### Limitations

Although this study enhances the understanding of the relationships among childhood neglect, peer attachment, the fear of missing out, and problematic smartphone use among young adults, it is not exempt from limitations. First, this was a cross-sectional study. Therefore, it cannot be used to establish causal relationships between the variables, such as peer attachment and problematic smartphone use. Also, a previous study found that peer attachment had a bidirectional association with Internet Gaming Disorder [41]. Second, the sample involved in this study only comprised college students from four public universities in the Jiangsu and Shanxi provinces in China. Therefore, it is not representative of other young people across the world. Third, this study relied on self-reporting for data collection, and this approach involves the risks of social desirability bias and recall bias [42]. Fourth, demographic data such as gender, grade and urban or rural students were used as control variables. However, other family/school factors were not controlled, such as parental attachment, class psychological environment.

### Directions for future research

Future research should be conducted to address the limitations mentioned above. First, future studies may involve longitudinal or experimental studies aimed at investigating the causal order of the relationships between childhood neglect, peer attachment, and the fear of missing out as they relate to problematic smartphone use among young adults. Second, future studies should focus on a more representative sample of young adults, not just college students, to expand the scope of the research design associated with this study. Third, to solve the limitations associated with the self-report approach, future studies should adopt objective measurement methods and collect data from multiple informants, such as parents, teachers, and peers. Fourth, more family/school factors should be controlled to validate the results of this study in the future study.

## Conclusion

In this study, we investigated the way in which peer attachment and the fear of missing out mediate the relationship between childhood neglect and problematic smartphone use among young adults. The results indicated that peer attachment and the fear of missing might have partial as well as sequential mediating effects on the relationship between childhood neglect and problematic smartphone use among young adults. Generally, the results of this study provide a novel perspective on exploring the relationship between childhood neglect and problematic smartphone use among young adults. Therefore, the results of this study might help in the improved understanding of the associated phenomena in the context of Chinese communities. The findings of this study imply that childhood neglect is related to different levels of peer attachment and the fear of missing out, which in turn affect the levels of problematic smartphone use among young adults.

## Data Availability

The datasets generated during and/or analysed during the current study are available from the corresponding author on reasonable request.
